# Improving quality of care and long-term health outcomes through continuity of care with the use of an electronic or paper patient-held portable health file (COMMUNICATE): study protocol for a randomized controlled trial

**DOI:** 10.1186/s13063-015-0760-8

**Published:** 2015-06-04

**Authors:** Marissa Nichole Lassere, Sue Baker, Andrew Parle, Anthony Sara, Kent Robert Johnson

**Affiliations:** Prince William Wing, St George Hospital, South Eastern Sydney Local Health District, Gray St, Kogarah, Sydney, 2217 NSW Australia; School of Public Health and Community Medicine, Faculty of Medicine, University of NSW, Level 2 Samuels Building, Samuels Ave, Kensington, Sydney, NSW 2033 Australia; St George and Sutherland Clinical School, Faculty of Medicine, University of NSW, Level 2 Clinical Sciences (WR Pitney) Building, St George Hospital, Short St, Kogarah, Sydney, 2217 Australia; Clinical Information Services, Prince of Wales Hospital, South Eastern Sydney Local Health District, Barker St, Randwick 2031, Sydney, Australia

**Keywords:** Personal health records, Electronic medical records, Health information technology, Randomized controlled trial, Pragmatic trial, Medical decision making, Medical informatics, Health services research

## Abstract

**Background:**

The advantages of patient-held portable health files (PHF) and personal health records (PHR), paper or electronic, are said to include improved health-care provider continuity-of-care and patient empowerment in maintaining health. Top-down approaches are favored by public sector government and health managers. Bottom-up approaches include systems developed directly by health-care providers, consumers and industry, implemented locally on devices carried by patient-consumers or shared via web-based portals. These allow individuals to access, manage and share their health information, and that of others for whom they are authorized, in a private, secure and confidential environment. Few medical record technologies have been evaluated in randomized trials to determine whether there are important clinical benefits of these interventions. The COMMUNICATE trial will assess the acceptability and long-term clinical outcomes of an electronic and paper patient-held PHF.

**Methods/Design:**

This is a 48-month, open-label pragmatic, superiority, parallel-group design randomized controlled trial. Subjects (n=792) will be randomized in a 1:1:1 ratio to each of the trial arms: the electronic PHF added to usual care, the paper PHF added to usual care and usual care alone (no PHF). Inclusion criteria include those 60 years or older living independently in the community, but who have two or more chronic medical conditions that require prescription medication and regular care by at least three medical practitioners (general and specialist care). The primary objective is whether use of a PHF compared to usual care reduces a combined endpoint of deaths, overnight hospitalizations and blindly adjudicated serious out-of-hospital events. All primary analyses will be undertaken masked to randomized arm allocation using intention-to-treat principles. Secondary outcomes include quality of life and health literacy improvements.

**Discussion:**

Lack of blinding creates potential for bias in trial conduct and ascertainment of clinical outcomes. Mechanisms are provided to reduce bias, including balanced study contact with all participants, a blinded adjudication committee determining which out-of-hospital events are serious and endpoints that are objective (overnight hospitalizations and mortality). The PRECIS tool provides a summary of the trial’s design on the Pragmatic-Explanatory Continuum.

**Trial registration:**

Registered with Clinicaltrials.gov (identifier: NCT01082978) on 8 March 2010.

## Background

The advantages of patient-held portable health files (PHF) and personal health records (PHR) have been discussed in the medical literature for many years [1–4], and include improved continuity of care, improved patient understanding of instructions and encouragement for patients to take an active role in maintaining their health. In one outpatient service the primary determinants of patient-held paper PHF (p-PHF) acceptance and use were physicians’ support of the process and actual size of the record, that is, the smaller the better [1]. Although there are some practical problems and ethical arguments for and against the use of patient-held records (ownership, privacy and confidentiality), research indicates that there are no substantial practical drawbacks, and that there are considerable ethical benefits to be derived from some form of shared record-keeping [2, 5–7]. Patients mostly perceive the PHR as a personal document for reference, while GPs perceive it as a management and communication tool [3].

Different types of paper-based patient-held records have been considered, including full copies of patient files, extracted summaries and censored summaries. In the era of information technology, new electronic computer-based applications for medical records have been developed and have enabled patient-held PHFs to be electronic-based. Patient-centered recording with use of medical data to promote effective clinical communication and cooperative care is considered one important goal [8–10]. Standards and practices are required to provide a framework for electronic patient-centered health care, with appropriate regulations for the storage and exchange of health-care data and a greater emphasis on professional information management skills. Two other important goals are process-integrated decision support integrated directly into systems to access current medical evidence resources, and comprehensive easily retrievable patient data for research and health-care reporting. If these can be combined with an effective and acceptable form of patient-centered recording, one would anticipate that with time, health-care outcomes would improve and costs would decrease.

Several models of patient-centered electronic health records have been developed. Top-down approaches are favored, particularly by public sector government and health managers. One approach involves a networked environment and unique patient and provider identifiers. In Australia, this approach included the Health Connect project (1999 to 2004) [11], which was not successfully implemented but was followed by the establishment of the National E-Health Transition Authority (NEHTA) in late 2004 [12]. One of NEHTA’s tasks was to develop standards to ensure consistent and accurate ways of electronically collecting and securely exchanging health information. This would allow medical data information exchange as well as a means for health-care research and for health statistics.

In a national approach to electronic health records, the electronic health record is seen as a tool for providing person-centered health care safely and efficiently in the modern information environment. The costs associated with the introduction of a national system are considerable. One overarching issue is the trade-off between ease of (appropriate) access and security. Interoperability, the ability to transfer and use information in a uniform and efficient manner across multiple organizations and information technology systems, is critical. An interoperability framework is a reference point for both existing and new e-health stakeholders, including planners concerned with the enabling role of technology in the delivery of health-care services, clinical informatics experts concerned with the meaning of information, enterprise and solution architects, as well as the providers and recipients of health care.

In 2009 another top-down approach was recommended by the Australian National Health and Hospitals Reform Commission: the Personally Controlled Electronic Health Record (PCEHR) system. The PCEHR is meant to be a secure electronic record of medical information that is stored and shared in a network of connected systems. Every Australian resident has been allocated a unique national identifier and every registered health-care provider has been allocated a unique national health-care provider identifier. Vendors are developing products that can upload information to the PCEHR. It is an opt-in system, not yet fully operational, and ethics and privacy concerns remain [13, 14]. Although launched in 2012, problems with roll-out and lack of meaningful use led to a review published in May 2014, which suggested extensive changes to the program [15].

Other approaches include systems developed by providers, consumers and industries that have developed local portals of patient-held or patient-controlled PHFs or PHRs. These are electronic applications through which individuals can access, manage and share their health information, and that of others for whom they are authorized, in a private, secure and confidential environment. Models of PHRs include stand-alone systems or systems integrated with health provider electronic medical records, PHRs carried on smart cards, on CD ROMS and more recently, on USB flash drives, or PHRs accessed through a secure web-based portal [16–18]. Web-based and other uploaded applications have been developed that allow patients to enter their own information into secure PHRs. International examples include HealthSpace, a website set up by the UK NHS [19], which closed operations in December 2012, and from industry [20], Google Health [21] (which discontinued its operations in 2011 and all data is being systematically deleted), and Microsoft HealthVault [22] and Dossia [23], both of which are still operating. The latter two either primarily target healthy and well-educated consumers or are directly linked to employer organizations or Health Maintenance Organizations (HMO), rather than being a tool for chronic disease management in patients with multiple co-morbidities. These web-based systems were promoted as a means of providing patients and providers with universal access to updated medical information. Most PHRs are not designed to be the primary record of the health-care system, but a prospective extract of core medical information (such as medical conditions, medications, results of investigations and procedures). Some systems are free and open source and claim to meet policy requirements of data encryption, secure access, authentication and authorization. One review of web-based systems reported limited functionality [18].

Whether top down or bottom up, the challenges in establishing IT systems in health care are many, including technical hurdles, administrative needs, legal requirements and resistance and/or inertia from a commitment to existing systems. The entire undertaking is predicated on an assumption of meaningful added-value attributed to the IT system compared to conventional systems. Testing this assumption and quantifying benefit are critical stages in the development of an IT system, and proof-of-concept during development will play a key role in either stimulating or discouraging further work.

Formal scientific assessments of added-value of electronic medical record support systems are often conducted in small networks of group practices, in a large single hospital or similar health-care organization and assess process outcomes such as degree of acceptability or satisfaction [24, 25]. Some support systems have documented reductions in mortality in before-after non-randomized settings [26]. Although such process assessments are important and necessary steps, and non-randomized assessments of interventions are important, an IT system with documented benefits in patient-relevant health outcomes in a randomized design would have a much stronger case for broad usage. There are randomized controlled trials in this setting [27–30]. Two randomized trials attempted to demonstrate benefit in short-term clinical outcomes, quality of life in oncology and symptoms scores in schizophrenia. Neither was successful, although both were arguably underpowered. Importantly, it was difficult finding reports of randomized trials of an IT health-care support system measuring important long-term outcomes of health care. One study in Australia that encompassed a variety of process measures failed to show differences comparing a computer-generated PHR with conventional records [27].

In 2005 we set out to evaluate a patient-held PHF carried by the patient on a USB flash drive. The pilot study design was a prospective non-randomized trial in patients with rheumatoid arthritis. The method of patient allocation to PHF was as follows: the first 25 % of recruited patients were allocated to no PHF (control group), the second 25 % were allocated to New South Wales (NSW) Health My Health Record, the third 25 % were allocated to p-PHF and the fourth 25 % were allocated to electronic PHF (e-PHF). Non-randomized allocation was conducted for this pilot because PHF development and availability varied, the project timeline was short (funding was available for only nine months) and because not all GPs had access to or used a computer. Patients of GPs without computers were not allocated to e-PHF. Halfway through the project patients in the no PHF arm were allocated to either p-PHF or e-PHF [31]. Both the newly designed paper-based and electronic files contained a core data set of information that included a directory of health-care providers, medical conditions, medications (current and past), investigations and visit summaries. This core data functioned as a subset of a more comprehensive electronic or paper-based medical record. It was structured to be patient and doctor-friendly and was not primarily a physician record. The PHF was updated by the doctor at each visit and could also be updated by patient between visits.

The e-PHF included audit information for each modification, including the computer host and login name identification of the doctor and visit date. The combination provided identification and non-repudiation for the patient data, and user’s ordinary authentication via the host computer operating system. The patient information was stored as separate files for each patient in the form of xml files encoded using a standard compression algorithm. Hardware and software were inexpensive and maintenance costs were small. The USB flash drive was robust, fast and stored large data files as well as all required software. The electronic file, an XML standard document, could import and export specified data, thereby reducing duplicate data entry, minimizing workflow interruption and facilitating synchronization [32]. Quantitative and qualitative methods were used in the evaluation of the project’s outcomes. This project was funded by the Commonwealth of Australia, Australian Musculoskeletal Quality Improvement Project.

In this pilot study, a consecutive sample of 105 patients with rheumatoid arthritis from three rheumatologists were asked to participate over a four-month-period, and nearly 80 % agreed after discussion with family and their GPs. The final sample was 76 patients, the average age was 63 years and the majority were female (78 %) and were born in Australian (74 %). The final GP sample size was 62. The study showed that the use of a patient-held PHF improved health-provider communication, patient and practitioner satisfaction and increased implementation of core set of quality indicators in arthritis [31]. Seven focus groups were also held, consisting of 43 patients and 12 GPs. Privacy and acceptability by both patients and health-care providers were evaluated in these focus groups and using self-report questionnaires. About 10 % of GPs did not wish to participate in the trial, some dissuading their patients from continuing, citing concerns regarding privacy and confidentiality. Most of these GPs had practices outside the catchment area of the health service that was conducting the project. Otherwise, the results were very encouraging [33]. A total of 80 % of patients would recommend a PHF to others, almost all liked to idea of carrying their own health data using a PHF and about half added information to the PHF. A minority (about 13 %) were often or sometimes concerned about privacy. Patients mostly perceived the PHF as a means of carrying information between health-care providers, and the majority of GPs said they would use it in the future [31].

In this pilot study we evaluated proof-of-concept practicalities that would be encountered in randomized controlled trial in Australia, including success of recruitment procedures, feasibility of subject compliance with tools and assessments, quality of the data collection forms and use in sample size estimations. We used this knowledge to design a larger randomized controlled trial of a paper and electronic-based patient-held PHF across all patient disease groups. The COMMUNICATE trial will assess the acceptability and long-term outcomes resulting from the usage of e-PHFs (carried by the patient on a USB flash drive) and p-PHFs in a population with high intensity use of medical services. The rationale is that use of PHFs provides a conduit of direct communication among health-care providers of important health-care information, and that this leads to better care and patient outcomes.

## Methods/Design

### Aims and objectives

This is a three-arm, randomized controlled trial that compares usual care (no PHF) to two patient-held PHFs: an e-PHF carried on a USB flash drive, and a p-PHF, in patients that are 60 years or older and who have two or more chronic medical conditions (see Inclusion criteria). The primary objective is to determine whether use of a PHF compared to usual care, over 48 months, reduces a combined endpoint of deaths, overnight hospitalizations and serious out-of-hospital events.

The secondary objectives are to determine whether use of a PHF over 48 months compared to usual care improves quality of life as measured by the EQ-5D [34, 35], and health status as measured by the Short-Form Health Survey SF-36 [36], and whether PHFs are acceptable in terms of user-friendliness, convenience and usefulness to patients and their health-care providers.

Additional objectives are to determine whether use of a PHF over four years compared to usual care improves delivery, quality and efficiency of health care by improved health literacy, reduction in medication errors, reduction in duplicative investigations, improved adherence to management plans, reduced health-care utilization and reduced costs.

The assigned intervention (e-PHF, p-PHF or no PHF) will be used for 48 months. Patients will also be followed up on for an additional 36 months beyond the conclusion of the randomized trial. This protocol is reported in accord with the Standard Protocol Items: Recommendations for Interventional Trials (SPIRIT) 2013 guidance for protocols in clinical trials [37].

### Methods: participants, interventions and outcomes

#### Design and setting

**Fig. 1:**
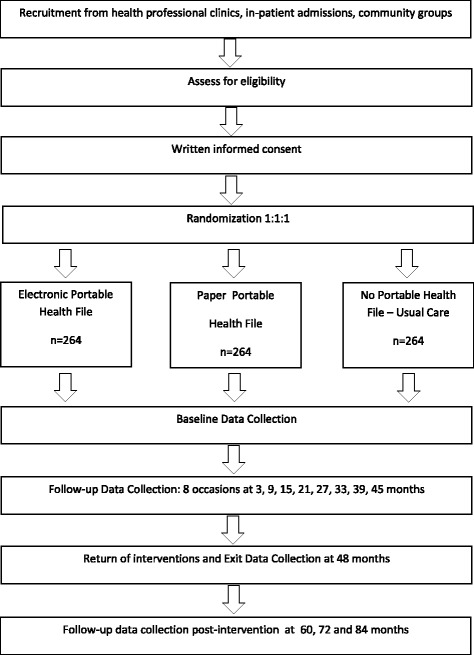
Flow diagram: the COMMUNICATE trial

This single-center pragmatic study is an open-label, randomized, parallel-group design. Patients will be randomized in a 1:1:1 ratio to each of the trial arms: the e-PHF added to usual care, the p-PHF added to usual care and usual care alone (no PHF). This is a 48-month trial and the assigned treatment (e-PHF or p-PHF) will be used for 48 months. Patients will also be followed up on for an additional 36 months beyond the conclusion of the randomized component of the trial. Patient trial data collection begins at three months post allocation of their intervention, and continues every six months until 45 months. At 48 months a detailed assessment data collection is collected. Following this trial subjects are monitored at annual intervals for a further 36 months (Fig. 1). The trial is conducted in the suburban regions of St George and Sutherland in southern Sydney, Australia.

#### Selection of patients

The trial population is enriched with patients who are high-intensity users of health care.

### Inclusion criteria

#### Demographic

1. Subjects must be of age 60 or greater.
2. Subjects living independently in the community; hostel care is acceptable, but patients that are not independent, that is, requiring full nursing home care are excluded.

#### Intensity of medical care

3. Subjects must have had six medical practitioner visits in the previous 12 months.
4. Subjects must have at least two of the following confirmed chronic diseases that require prescription oral or parenteral drug treatment or surgery, and require at least annual specialist consultation: cardiovascular, respiratory, endocrine, renal, neurologic, gastrointestinal, hepatic, genitourinary, hematologic, infectious, rheumatic, inflammatory, immunologic or neoplastic diseases.

#### Computer resources of GP and of specialists

6. Subject’s GP must have access to a computer with a Windows operating system during the consultation visit.
7. Subject must have had at least two medical specialists, at least one of whom has access to a computer during the consultation visit.

#### Ethical

8. Subjects must be able to understand the purpose of the trial and provide full and valid informed consent.

### Exclusion criteria

#### Medical

1. Subjects with a life expectancy of less than 12 months.

#### Other

2. Subjects who are unable to carry a p-PHF or e-PHF and have no caregiver willing and able to do this on their behalf.
3. Subjects who are mentally unable to provide valid informed consent.
4. Subjects who are not independent in the community, that cannot mobilise to see a health-care provider or require full nursing home care.

### Subject recruitment

Health-care providers will be contacted directly about the trial, and patient information brochures and posters will be provided in practice waiting rooms, hospital wards, outpatient clinics and local community groups. There will also be information sessions through specialist societies, divisions of general practice and through the local media. Several methods of subject recruitment will be used to ensure that patients who are high users of health services participate: recruitment from health provider practices, both general practice and specialist practice, and recruitment from inpatients at St George and Sutherland public hospitals. Potential subjects will be given a folder containing information about the study. This includes a detailed information sheet, an informed consent form and material to give to their family and/or health-care provider. The study personnel will clearly explain that because the trial is a randomized trial, whether the patient receives a PHF is determined by a random process. A log is kept of all patients that meet the eligibility criteria. A patient is considered to be enrolled in the study upon completion of the informed consent process and have been randomized.

After the patient has consented, their GP will be contacted by the study investigators and a time organized to provide information about the project, demonstrate the tools and, if a patient was randomized to the e-PHF, load the software and authenticate the GP. After the patient has consented, they will be interviewed and a baseline medical history will be obtained, using their medical records where available. This history includes all current and previous medical conditions, procedures, medications and alerts. The patient’s other health-care providers, such as their specialists, will not be routinely contacted, except when asked to complete a baseline health-care provider questionnaire and for the authentication process. These health-care providers will be approached by the participating patients during their routine appointments.

### Interventions

#### Electronic portable health file

**Fig. 2:**
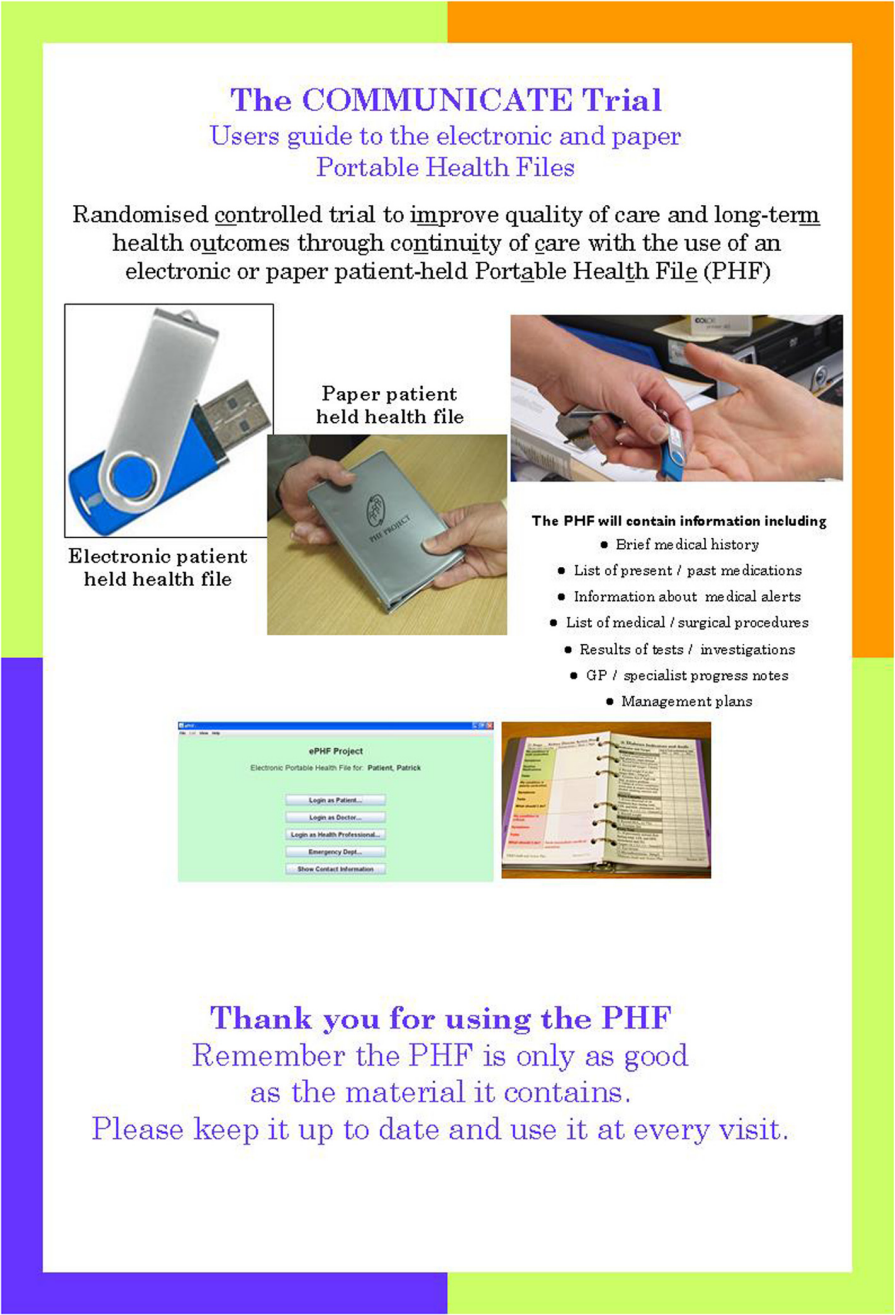
The electronic and paper portable health files

The e-PHF concept and software were developed to implement the objectives of this study. The e-PHF is stored on a USB flash drive (Fig. 2). Scripts for the installation process and initiation of the antivirus software are included at the front-end of the program The software is written in Java, uses Eclipse [38] (an integrated development environment) and requires a Java Runtime [39] to execute, which in principle allows it to run on any of the major platforms. The patient information is stored in separate files for each patient in the form of xml files encoded using a standard compression algorithm. The e-PHF includes encrypted XML in an extensible schema, with click and type interface to facilitate functions such as doctor authentication, audit trail, non-repudiation and saving of changes built-in and automated. The file is read-only without the USB flash drive. Therefore the e-PHF cannot be edited unless the USB flash drive is connected to the health-care provider’s USB port with the associated audit trail. This feature provides synchronization among all users. Software information includes audit information for each modification, including the computer host and login name identification of the doctor and date. The combination provides identification and non-repudiation for the patient data, and user’s ordinary authentication via the host computer operating system.

Several levels of password protection and doctor authentication are required. The e-PHF runs from the USB flash drive which also includes antivirus software. The USB flash drive also carries the software allowing it to be installed on computers of any new health-care provider once the patient has provided permission. Written as well as online information regarding the project and installation is provided, as well as a free 1800 telephone support number. Information regarding the study is incorporated on a tag that is attached to the USB flash drive and the 1800 telephone number is engraved onto the USB flash drive. Self-installation is encourage to evaluate the projects sustainability, and a record will be kept of how many health-care providers self-installed the software from the patient’s USB flash drive versus having the project support team install the software.

Once the patient has securely entered their password and the e-PHF application launches, the health-care provider has the opportunity to become an authenticated user. This requires the health-care provider to contact the study personnel on an 1800 support number. Once the health-care provider generates their own password, and after the study personnel authenticates the health-care provider’s identity, the health-care provider is given an authentication key. This authentication process only can proceed if the USB flash drive is connected to the host computer. The authentication key creates synchronized a record of the entire e-PHF on the host computer to allow the provider to read (but not write to) the e-PHF record after the patient has left the consultation, without requiring connection of the patient’s USB flash drive. However, security is still enforced, as the health-care provider must enter their password to launch and read the patient’s read-only record.

If the USB flash drive is lost, the data is encrypted and cannot be accessed without the patient’s password. A new USB flash drive can be repopulated with the entire e-PHF, once password protocols are met, and the patient takes the new USB flash drive to the last authenticated user. There are a number of other features of the e-PHF, designed to improve accuracy and acceptability, which are not provided in this protocol.

#### Paper portable health file

**Fig. 3:**
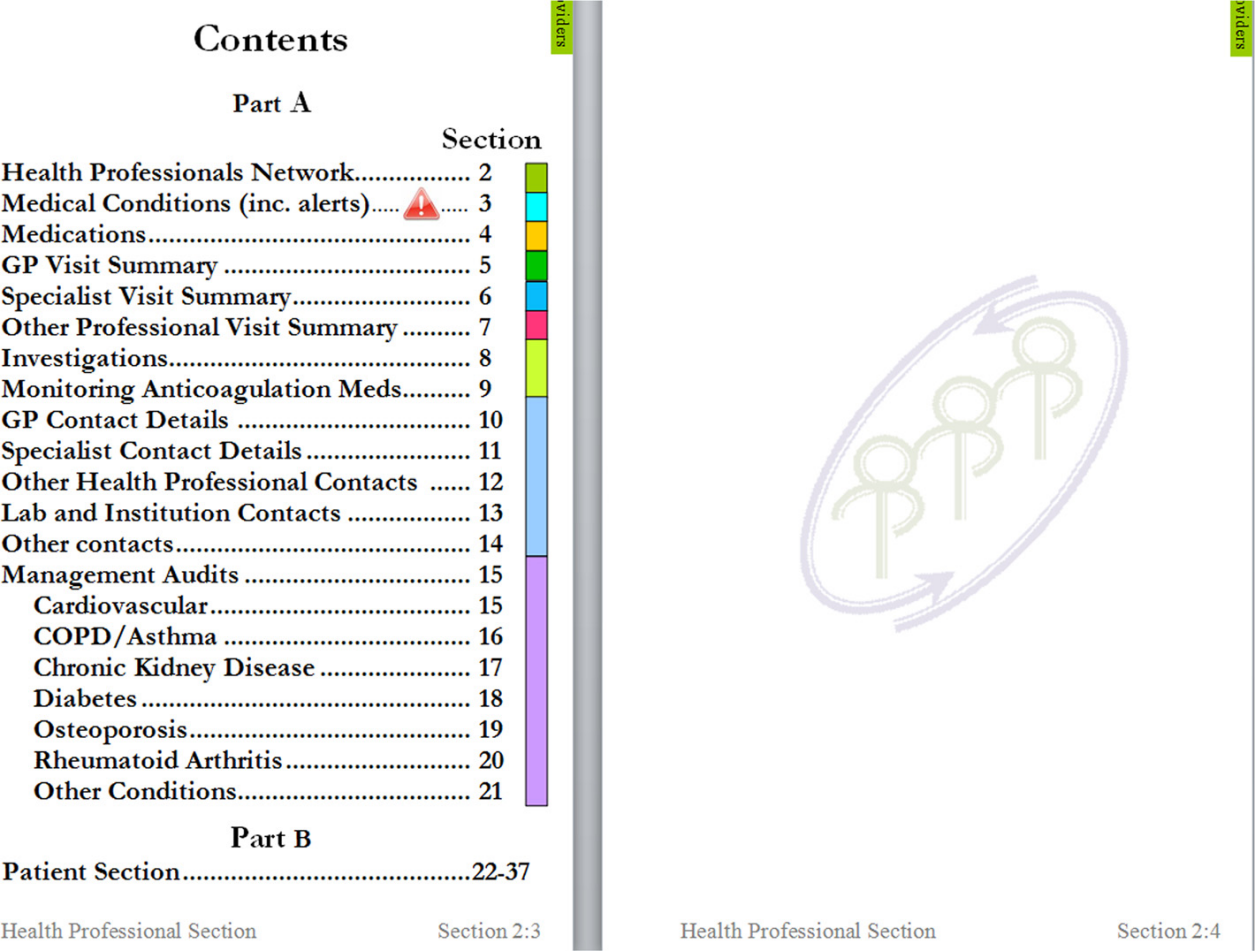
Excerpt from contents of the paper portable health file

The p-PHF was designed to implement the objectives of the study. It is a color-coded, 280-page, A6 ring-bound book in 39 sections that contains general information, medical and other health-care related information. It contains a health-care provider section in part A, and a patient section in part B (Figs. 2 and 3). As it is paper the book is not secured. If the book is lost, then only the pages that were scanned at the six-monthly intervals by the study personnel can be repopulated in another p-PHF, unless a health-care provider or the patient has copied the pages in the intervening period.

### Trial arms

#### Arm one: electronic portable health file

Arm one is the e-PHF added to usual care. The e-PHF will be kept by the subject (or caregiver) and taken to health provider visits. At each visit the health-care provider has the opportunity to read the contents of the e-PHF. The health-care provider then has the opportunity to write to the e-PHF. The information that is recorded is information that is deemed relevant by the health-care provider. It can be a record of the entire visit or a summary of the visit. The current visit information can subsequently be printed, exported or copied and pasted into another file. Information that the health-care provider believes the patient may not want disclosed to other health-care providers is discussed with the patient, and a decision is made whether it should be recorded in the e-PHF or not. The medical information that can be recorded includes recent and past medical problems and conditions, examination findings, investigations requested and their results, current medications, medication changes, any adverse events, condition management plans and clinical audits of the processes of care derived from best practice guidelines. Patients can also enter this information in their patient section. Patients have access to read all sections of the e-PHF, but can only write to the patient section.

#### Arm two: paper portable health file

Arm two is the p-PHF added to usual care. The p-PHF intervention in content is similar to the electronic counterpart, but is paper-based within an A6 spiral bound book.

#### Arm three: usual care

Arm three has no PHF and is usual care only.

#### Additional procedures

All subjects randomized to either the e-PHF or p-PHF will receive a PHF pre-populated with a summary of their baseline medical history, including past and current medical conditions, current medications and alerts. This information is obtained after an interview with one of the study’s medical investigators, who is a medical doctor and who has access to the GP, specialist and hospital medical records for verification. Once the PHF has been prepared with the patient’s medical information, the patient is asked to verify that the information is correct. Patients randomized to usual care also undergo the same processes, but the information is not used to populate a PHF and is kept for analysis purposes.

All patients also complete a baseline questionnaire that includes items on demographic information, social and lifestyle information, experience with computers, quality-of-life information, health-care utilization, perceptions about personal health files, health-care communication, health-care satisfaction and health literacy.

### Outcomes

#### Primary outcome

The primary outcome is a combined endpoint of the total number of important clinical events, defined as: all overnight hospitalizations, all serious out-of-hospital events and death. Validation of hospitalizations or out-of-hospital events occurs by subject interview, and health-care provider and hospital records as close to real time as is possible. In addition, record linkage with Medicare Australia, Department of Veterans Affairs, Australian Institute of Health and Welfare and NSW Health during the study is another source of valid information on hospitalizations, health service utilization (including a record of all medical encounters) and deaths. Furthermore, an Independent Adjudication Committee (IAC) determines which out-of-hospital events are serious, and are therefore included in the primary combined outcome.

#### Secondary outcome

Secondary outcomes include patient interview and questionnaire self-report data on quality of life (SF-36 and EQ-5D), and whether PHFs are acceptable in terms of user-friendliness, convenience and usefulness to patients and their health-care providers. Additional outcomes include adherence to management plans, duplicate investigations and adverse drug reactions. Subjects will be asked to complete questionnaires at baseline and at regular intervals over the course of the trial (Table 1). The questionnaires include items on health literacy (the short Test of Functional Health Literacy Assessment (TOFHLA) [40, 41] and the Single-Item Literacy Screener (SILS) [42]), experience with information technology, acceptability of and satisfaction with the e-PHF or p-PHF, measures of functional status (SF-36) and utility (EQ-5D), health service utilization, all health-related events, satisfaction with medical care, perception of continuity of care, and adverse events. Additional information will be recorded from the p-PHF and e-PHF to evaluate its real-time use.

**Table 1:** Schedule of assessments

Time point (months)	T_0_	3	6	9	12	15	18	21	24	27	30	33	36	39	42	45	48	60	72,84
**PATIENTS**																			
Demographic information	X	X		X		X		X		X		X		X		X	X	X	X
Information technology literacy	X																X	X	X
Health literacy	X																X	X	X
Health and Lifestyle	X																X	X	X
Satisfaction with Health Care	X																X	X	X
Quality of life/SF-36	X	X		X		X		X		X		X		X		X	X	X	X
Health Status/Utility/EQ-5D	X	X		X		X		X		X		X		X		X	X	X	X
Health care utilization	X	X		X		X		X		X		X		X		X	X	X	X
Satisfaction with Health Professional Communication	X																X	X	X
Acceptability of PHF^a^	X																X		
Patient Compliance with PHF^a^		X		X		X		X		X		X		X		X	X		
Health professionals compliance with PHF^a^		X		X		X		X		X		X		X		X	X		
Hospitalizations and Deaths^b^		X		X		X		X		X		X		X		X	X	X	X
Serious out-of-hospital events^b^		X		X		X		X		X		X		X		X	X	X	X
Medications use		X		X		X		X		X		X		X		X	X	X	X
Duplicate Investigations		X		X		X		X		X		X		X		X	X	X	X
Adverse events		X		X		X		X		X		X		X		X	X		
Retrieval of PHF^c^		X		X		X		X		X		X		X		X	X		
Exit interviews																	X		
**HEALTH PROFESSIONALS**																			
Demographic information	X																		
Acceptability of PHF	X																		
Satisfaction with Health Professional Communication	X																		
IT experience	X																		
Compliance with PHF by patient																	X		
Compliance with PHF by health professionals																	X		
Adverse events																	X		
**HEALTH PROFESSIONALS’ MEDICAL RECORDS**																			
Comparison of PHF with health professional medical records^d^																	X		
**RECORD LINKAGE – DATABASE** ^**c**^																			
Deaths																	X		X
Overnight Hospitalizations																	X		X
Day Only Hospitalizations																	X		X
Emergency Department Visits																	X		X
Health care utilization- health professional visits																	X		X
Health care utilization-prescription medications																	X		X

^a^Health Professional compliance will be done only for his/her patients enrolled in the trial. Subject acceptability, satisfaction and compliance will be done on all PHF subjects. Subject compliance will be undertaken by questionnaire and when the interventions are recalled for data extraction. Control no-PHF subjects will also be contacted and given the opportunity to discuss trial-related activities to reduce bias

^b^These assessments will be elicited from the GP and other providers, from subjects or their primary health care givers in real time during the trial. It will be validated by medical record data where possible, then adjudicated by the blinded adjudication committee

^c^Record linkage with NSW State and Commonwealth databases when the last subject has exited the randomized component of the trial and again 3 years after the last subject has exited the trial

^d^GP compliance evaluates data from the medical record with data recorded on the electronic PHF or in the paper PHF

Health-care providers will also provide information regarding their experience with information technology, acceptability of and satisfaction with the e-PHF or p-PHF, uptake of management plans, medication errors, duplicative investigations, health service utilization and adverse events by interview and questionnaire at baseline and at 48 months.

The PHF will be retrieved from the subject at three months and then every six months, and as per the assessment schedule for collection of process measures and clinical outcomes. The p-PHF pages are scanned and the e-PHF data is copied. In the event of loss of the p-PHF, a replacement notebook will be provided will be populated using the patient’s stored information. In the event of a lost USB flash drive, a copy of the latest file can be retrieved from the computer of the last authenticated health provider visited with their e-PHF. If a PHF has been lost this event is documented as an adverse event, but not a serious adverse event.

### Safety, adverse events and risk assessment

An adverse event is defined in the International Conference on Harmonisation (ICH) Guideline for Good Clinical Practice as ‘any untoward medical occurrence in a patient or clinical investigation subject administered a pharmaceutical product and that does not necessarily have a causal relationship with this treatment’. In this trial, any known breach of PHF security, accuracy and confidentiality, whether intentional or unintentional will be considered an adverse event. A serious adverse event will be considered to be where these breaches lead to physical or emotional harm, as perceived by the participant. All adverse events are adjudicated by the committee. Disclaimers are included in the p-PHF and e-PHF regarding the medical information, and users are cautioned that there is the possibility of error or inaccuracy. The disclaimer also states that the user’s professional and clinical expertise and judgment are required at all times when using the information contained in the PHFs. Adverse events data, including seriousness of the adverse event, is collected with every questionnaire and during retrievals.

### Methods: assignment of interventions

#### Sequence generation and allocation concealment

Subjects will be randomized to one of three arms using computer-generated random numbers. The allocation sequence is concealed as randomization is via a central telephone using an Interactive Voice Response System ((IVRS) maintained at a separate organization). Factors for stratification are: aged 60 to 74 years or over 75 years; private medical insurance or no private medical insurance and hospitalization in the previous 12 months or not. Once the study manager confirms eligibility and obtains informed consent, the study manager calls the IVRS and after entering the strata factors, is given the randomly assigned intervention by the IVRS.

#### Blinding

Once the subject is enrolled in the trial they will be allocated a unique subject identifier, which is a six-digit number without any other identifier (subject ID). Any further documentation associated with the subject will only be identified by this number. Only persons directly related with this trial will have access to the database or stored records. The nature of the intervention does not permit blinding of either trial participants or health-care providers. Therefore, there will be potential for bias in trial conduct and potential for ascertainment bias in the determination of important clinical outcomes and quality of life. To reduce clinical outcome ascertainment bias, a blinded IAC will be used to make the determination which out-of-hospital events are serious. The other primary outcomes, mortality and all overnight hospitalizations, are objective outcomes and are not subject to ascertainment bias. Data on all hospitalizations, same-day hospitalizations, 28-day readmissions and deaths are administrative outcomes, coded and collected by the clinical information systems of public and private hospitals within the state of NSW. Questionnaires that collect information on secondary outcomes are mailed to all trial subjects without any assignment identifiers. All statistical analyses unrelated to acceptability of PHF and compliance of use are masked to intervention allocation via a separate data set.

### Methods: statistical analysis plan

All analyses will include all subjects randomized regardless of whether or when they may have withdrawn from the study. Therefore, for the primary and secondary objectives, the analysis will follow intention-to-treat principles. However, a completer’s analysis which is a comparison based on random allocation of subjects with complete data at the end of the trial, will also be undertaken as well as an as-treated complier’s analysis, which is based on the actual intervention and for how long it was utilized. The completer’s analysis and the as-treated analyses are exploratory analyses only.

#### Hypotheses

The primary hypothesis is that addition of a patient-held PHF to usual care results in fewer important clinical events using a combined endpoint of deaths, overnight hospitalizations and serious out-of-hospital events compared to usual care. The secondary hypothesis is that addition of a patient-held PHF to usual care improves quality of life and health status.

#### Primary statistical analyses

There are two co-primary analyses because this is a three-arm, randomized controlled trial: e-PHF versus usual care and p-PHF versus usual care. Both analyses use the combined endpoint of deaths, hospitalizations (excluding day-only admissions) and adjudicated serious out-of-hospital events, and both use an adaptation of the Andersen-Gill formulation [43] of the proportional hazards model in order to capture possibly correlated events in the same subject. A robust variance estimate [44] will be used that takes into account the possibility of correlation of risk of multiple events within a subject by including an additional time-dependent covariate indicating whether the event was the first or subsequent event of its kind. The level of significance for each analysis is 0.025. This is an intention-to-treat analysis.

#### Secondary analyses

Secondary analyses with be comparison of quality of life and health status, and incremental cost per quality-adjusted life-year with confidence interval by Fieller’s theorem [45, 46]. In analysis of quality of life, missing data will be addressed by imputation with sensitivity analysis.

#### Other analyses (exploratory)

1. Analyses as for the primary analysis but adjusted for study completion after 48 months of treatment (completers analysis);
2. Analyses as for the primary analysis but adjusted for study completion after 48 months of treatment and adherence with interventions (compliers analysis);
3. Subgroup analysis of each of the composite endpoints: hospitalizations, serious out-of-hospital events and deaths;
4. Subgroup analysis of day-only hospitalizations;
5. Analysis of patient acceptability;
6. Analysis of health-care provider acceptability;
7. Qualitative exit interviews and
8. Subgroup analyses of primary and secondary endpoints adjusted by covariates: age, health literacy, comorbidity status and computer and information technology literacy.

#### Additional analyses

9. Health service utilization and health-care costs;
10. Medication errors, duplicative investigations;
11. Clinical workflow;
12. Management plans uptake and documentation;
13. Health literacy;
14. Information technology and computer expertise and
15. Adverse events.

#### Sample size and power calculations

Hospitalizations are likely to dominate events in the composite endpoint of hospitalizations (excluding day-only hospitalizations), serious out-of-hospital events and deaths. Therefore hospitalization data were used to power the study, with the aim to detect a 25 % reduction in hospitalizations. The sample size and power calculations for the study were based on the Fisher’s exact test, as this is a conservative analysis and enables use of the Australian Institute of Health and Welfare data (AIHW) [47] that indicate a yearly risk of hospitalization of 62 % for women and 78 % for men aged 66 to 69, and 80 % and 115 % for women and men aged 70 to 74, respectively, using hospital separation data from 2003 to 2004. The AIHW data are for all hospitalizations. This study excludes day-only hospitalizations in the composite endpoint, but includes serious out-of-hospital events and deaths. Overall, we estimated that at least 70 % of the subjects in the control arm would have at least one event (either an overnight hospitalization, a serious out-of-hospital event or death) throughout the 48-month duration of the trial. With these assumptions, 80 % power and a 0.025 level of significance, to detect a 25 % reduction in hospitalizations requires 158 subjects per arm for analysis by a Fisher’s exact test of proportions. In order to provide for the loss of power due to patients discontinuing (at the most conservative, assuming a 40 % non-differential dropout from the intervention arms), the trial requires 792 subjects, or 264 subjects per arm. Regarding local feasibility, in 2008 there were 11,421 unplanned admissions via the emergency department at our institution (St George Hospital) alone for patients aged 60 years or older. The entire study area of southern Sydney has several public and private hospitals.

### Ethics and governance

Informed consent will be obtained from each participant. The project has been approved by the South Eastern Sydney Local Health District Human Research Ethics Committee - Southern Sector (EC00135) reference number: 06/124 Lassere (approval letter dated 8 December 2006). A recent amendment was approved (2 September 2014) to conduct exit interviews to obtain further qualitative data regarding the project. An IAC that includes a neurologist, a cardiologist, a rheumatologist and an infectious disease physician will evaluate all reported serious out-of-hospital events to determine whether they consider the event was a serious out-of-hospital event. All adjudications are undertaken blind to randomized arm status. The committee includes the Chief Investigator (ML) and project manager (SB) for administrative purposes only.

## Discussion

The PHF protocol is, to our knowledge, the first example of a randomized study of an electronic assist to medical records that tests whether use improves the rate of important patient-relevant outcomes, overnight hospitalizations and deaths (plus medically important out-of-hospital events, anticipated to be rare). This framing of the hypothesis (reduction in hospitalizations and deaths) is crucial because past experience has not infrequently found that clinical studies using intermediary clinical outcomes or biomarkers often mislead when tested in subsequent outcome studies [48]. Our use of patient-relevant outcomes is of further importance given the extensive enthusiasm surrounding expected gains offered by contemporary information technology. A pivotal study in this area must be firmly based in unequivocally convincing outcomes. Additionally, what is needed is not only a test of the e-PHF compared to usual care, but to anchor this result with a p-PHF result for comparison; thus, we employ a three-arm design.

When we designed this study, there was substantial disenchantment with ‘big brother’ perceptions surrounding medical records stored centrally or in web-based systems, sentiments reflected in feedback in our pilot study and indicated by the failure of HealthConnect in Australia and similar systems elsewhere. Thus, we made the decision to empower the patient by having him or her be the holder of their PHF; it is the patient’s responsibility to take the PHF to their respective providers. In parallel, we canvass a very large portion of GPs in the relevant areas to solicit their participation. Implicit in this design is the need for compliance by both patients and providers in following the protocol. We entertained a fourth study arm, a web-based portal, but could not pursue this opportunity because of additional costs involved in terms of both web-based expertise and increasing trial size to accommodate additional comparisons.

Strictly masked treatment allocation (by remote computer) is employed. A variety of trial conduct features were used to limit bias present because full blinding was not feasible in this trial. Firstly, the primary endpoint was made objective (overnight hospitalization and death) and will be ascertained regardless of whether a patient has withdrawn from the study. Medically important serious out-of-hospital events are expected to be rare and will all be independently adjudicated. Because the most important secondary endpoint, quality of life, may be prone to ascertainment bias, we were particularly attentive to balancing patient interactions and contact across all three arms regarding the six-monthly data collection through questionnaires of quality-of-life measures, PHF acceptability and health service utilization, and periodic telephone inquiries to confirm events such as hospitalizations, out-of-hospital events and adverse events that may need adjudication. We are also attempting to obtain post-discontinuation quality-of-life measures and interviews in patients choosing to drop out of the trial. Although study personnel clearly explain that the study is a randomized trial and whether the patient receives a PHF or not is determined by a random process, patients may nonetheless agree to be recruited hoping to be randomized to a PHF arm, and later withdraw consent and drop out of the trial if they are randomized to usual care.

Possibly, a tendency to better health attention generally for the patients in the PHF arms could skew the hospitalization rate in either direction. However, merely undertaking this trial will yield a wealth of experience with the electronic assists to medical records, including a cross-section of older patients with multiple comorbidities, and high-end consumers of health services and their acceptance to carrying their medical information, their interactions with health-care professionals and systems and how to study this. Finally, it is recognized that any clear result, whether positive or negative, from this trial will provide hypotheses for the future, as the technology used will be antiquated in a short time.

It is recognized that studies of this size are underpowered to detect mortality differences alone. AIHW data [47] show a mortality rate of 0.95 % and 1.68 % for women aged 65 to 69 and 70 to 74, respectively. The data for men are 1.72 % and 2.90 %, respectively. Assuming a population of equal women and men and equal 65 to 69 and 70 to 74 age groups, the four-year mortality rate is 7.25 %. To detect a 25 % mortality reduction, assuming 80 % power and a 0.05 level of significance, 2,947 subjects per arm would be needed. The study could not recruit these numbers; therefore the trial was designed with a composite endpoint of overnight hospitalizations, serious out-of-hospital events and death as its primary endpoint. Neither endpoint on its own would have sufficient power for a subgroup analysis. Our sample size calculation was based on hospital separation data from 2003 to 2004, which was the most recent data available at the time we first planned the study. Hospital separation data from 2007 to 2008 showed an increase in overall separations to public hospitals of between 12.9 % and 28.8 % for those aged 65 years and older.

Randomized trials have previously been described as either primarily efficacy trials or primarily effectiveness trials. Schwartz and Lellouch in 1967 [49] described these two design approaches as explanatory or pragmatic trials. Generally, interventions are first evaluated under ideal conditions to evaluate a causal question and ensure internal validity of the results. However, these interventions are shown not to be applicable in the real-world setting: they are said to lack external validity, otherwise known as generalizability. Effectiveness or pragmatic trials attempt to address this gap, and recently the Consolidated Standards of Reporting Trials (CONSORT) statement has been extended [50] to improve the reporting of pragmatic trials. Two tools have been developed to help trial designers: the Pragmatic-Explanatory Continuum Indicator Summary (PRECIS) tool [51] and the Reach, Effectiveness, Adoption, Implementation and Maintenance (RE-AIM) framework [52].

**Fig. 4:**
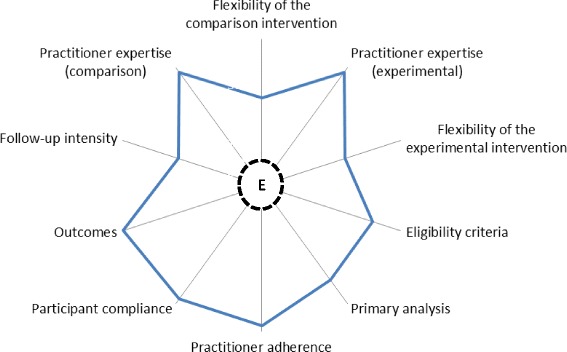
Pragmatic-explanatory continuum indicator summary of the COMMUNICATE trial

Pragmatic studies of PHRs are generally non-randomized [53] or employ cluster randomization [54], options that simplify the study and possibly improve participant adherence, but risk explanatory validity. In our study we have attempted to balance efficacy and effectiveness, and using the PRECIS tool, we provide a summary of where our trial sits on the Pragmatic-Explanatory Continuum (Fig. 4). The eligibility criteria include participants that are more likely to be responsive to the intervention as these are higher risk individuals because they are older and have more comorbidities, but there are few exclusions in that all such individuals are enrolled unless life expectancy is less than 12 months and there are cognitive or psychiatric reasons why consent informed cannot be provided. There is some flexibility regarding how the practitioners in the experimental and comparison arms apply the interventions or practice usual care. No minimum criteria are set except that the patient’s GP must have access to a Windows computer during the consultation, and that at least one of the specialist health-care providers also has access to a Windows computer. The primary barrier is that practitioners that use Apple products and software, or Linux operating systems cannot take part.

There is formal follow-up of participants to determine serious out-of-hospital events and obtain quality of life and other data, but the primary endpoint is a composite endpoint of hospitalizations (excluding day only), deaths and serious out-of-hospital events, and the former two are obtained from administrative registries. The study has been designed so that the primary outcome is clinically important to patients, caregivers, health-care providers and other health care-related personnel. Participant compliance to the prescribed intervention and practitioner adherence to the protocol are both unobtrusive, although they are measureable at the end of the trial. The primary analysis includes all patients regardless of compliance and practitioner adherence, so that a determination of whether the intervention works under usual conditions is determined. Unlike many other health-care system pragmatic trials, which are cluster randomized, we made a decision to randomize individual patients to the intervention, which provides a greater causal relationship and is more in keeping with an explanatory trial in our setting.

There is considerable evidence that patients like the PHF, believe it is useful and feel more in control of their care when they have access to it. These are important outcomes. The fundamental question is whether use of a PHF is acceptable enough short term to enable demonstration translation into important patient outcomes. It may well be that in the near term, physician visits and even hospitalizations may increase due to attention to aspects of chronic diseases previously overlooked. One mechanism by which we believe access to a PHF will result in changes to patient outcomes and processes involves the use of multiple medications among an older patient population with complex health problems. However, the important question is whether a PHF and any accompanying increase in intensity of care will translate into later benefits in morbidity, as measured by significant hospitalizations, which is probably the most feasible overall capture of morbidity. Only with a properly designed trial using randomization can one obtain a valid demonstration of a reduction in morbidity and in mortality. However, to date this has not been easy to demonstrate, and successful demonstration may vary with exactly what benefit is being considered and, as importantly, what time course is being used. Our proposal addresses both trial duration and size in a randomized setting with important clinical outcomes.

## Trial status

The COMMUNICATE trial began patient recruitment in March 2010 and we anticipate that recruitment will be completed in September 2015.

## Abbreviations

e-PHF: Electronic Portable Health File
EQ-5D-5 L: EuroQoL
IAC: Independent Adjudication Committee
IVRS: Interactive Voice Response System
NEHTA: National E-Health Transition Authority
PCEHR: Personally Controlled Electronic Health Record
PHF: Portable Health File
PHR: Personal health record
p-PHF: Paper Portable Health File
PRECIS: Pragmatic-Explanatory Continuum Indicator Summary
SF-36: Short-Form 36
